# A Case of Lead Encephalopathy

**Published:** 1909-06

**Authors:** J. Angell James


### A CASE OF LEAD ENCEPHALOPATHY.

BY

J. ANGELL JAMES, M.R.C.S., L.R.C.P.

---

I AM recording the notes of this interesting case for several reasons. It is a most uncommon condition. Out of 2,448 cases of plumbism reported during the last four years under the Workshops and Factories Act, only 84 were cases of encephalopathy; and Janquerel, the French physician, in his clinic reports 1,217 cases of colic, 101 of palsy, and only 72 of encephalopathy, of which latter 16 died, probably a small proportion.

It is a most serious condition, in which alarming symptoms may develop rapidly, and in which the diagnosis is not always clear, unless there is a definite history or unmistakable signs of lead. The cause in this case is unique. I have written to the Chief Inspector of Factories, and I cannot find that a similar case has been reported before.

This was a case of a young woman, aged 16—(young women are more susceptible to the action of lead than other people, and the poisoning in them more often takes the encephalic form)—who had been for the last two years "an artist colourist," an industry that has not been worked in England long, but has been in existence in Paris and Vienna for some years.

The process consists in painting the different colours on black and white prints, and is done by young girls from copies. The pictures when finished are sent abroad for sale for post cards, almanacs, etc. This particular girl was rather skilled, and for the last twelve months had been working with a particular white paint called "Blanc d'argent" (made by a Paris firm), and containing lead carbonate. The other white paint used, "Chinese white," contains zinc oxide. The peculiar effect of the former is a silvery appearance, it stands out in contrast to the rest of the picture, and adds much to its effectiveness, and is put on after the painting is done. In this case the poison was introduced into the system through the mouth, the girl having a habit of sucking her brush to get a better point.

The symptoms were most interesting and unusual. For some months prior to the time when I first saw her she had been in indifferent health—headache, sickness, constipation, a bad taste in the mouth and lassitude were complained of. Her temperament changed also: instead of the lively, bright girl she used to be, she became restless, irritable, nervous, did not like to be left alone and frequently suffered from nightmare.

I saw her first on October 29th, 1908. A strong, well-developed girl, a sallow tinge about the complexion, which was naturally bright, a very marked metallic odour about the breath, a distinct blue line along the gum, the teeth were of good colour and well kept. She had spent the last three or four days lying down, during which time there had been no action of the bowels and almost incessant vomiting; the vomit was yellow in colour and had a feculent odour. Pulse 75, temperature normal, there was slight retraction of the abdomen and a little tenderness; in fact, all the symptoms pointed to a diagnosis of plumbism.

The constipation was overcome by means of enemata, the first was not very successful, but much better results followed next day, and the treatment was continued for the next three or four days. Although the bowels were acting the vomiting continued; the only thing that could be retained was a little milk and soda. She was very restless, and would not be left alone a moment; had very little sleep.

On the fourth day she became drowsy, and complained of intolerance of light. This was followed after some hours by a general epileptiform convulsion, which lasted about five minutes, and again in three or four hours' time by another convulsion, more severe in character and lasting longer. There was no return of consciousness after the first fit. She remained practically unconscious for the next twenty-four hours, during which time she was seen by Dr. J. R. Charles.

She lay on her back and side with her head retracted and her legs drawn up. She resisted attempts to examine her. All her reflexes were present and slightly exaggerated; pupils were equal, rather dilated and sluggish; urine was passed involuntarily; breathing rather irregular; pulse 66; temperature slightly raised, and remained so for several days, but never reached 100 F. On the following night she had another convulsion, which was less severe. The unconsciousness remained more or less complete for another twenty-four hours, during which time she could be occasionally roused to take a little nourishment. She was very restless, frequently groaned, and vomited at intervals, after which she slowly improved, and symptoms gradually passing away, she was able to get up in a month, and was practically well at the end of three months. At the present time she has no symptoms of any kind.

*Digestive System.*—The vomiting, which was so incessant at first, disappeared at the end of a fortnight. The constipation was more difficult to overcome.

There was only the single blue line (Burton's) on the gum.

*Urinary System.*—There was a little albumen at first, but that had disappeared by the end of a fortnight. Urine was passed involuntarily from the commencement of the first fit for the next three or four days.

*Menstruation.*—There was complete amenorrhœa; menorrhagia is the usual condition.

*Limbs.*—There was a good deal of pain complained of, especially in the legs, and painful cramp in the calf muscles, which seems to be more common in young people in plumbism than older people. These symptoms lasted two or three weeks. When she first got about there was a slight weakness of the right leg, and the foot was dragged in walking, and slight difficulty at first with the left hand in playing the piano. No paresis otherwise of the muscles of the limbs.

*Nervous System.* — The headache was severe, especially frontal and occipital, with retraction of head and stiffness of neck, the pain at the back of the head and neck being greatly exaggerated on any movement, suggesting some meningeal mischief, and confirming the theory of Mosny (of Paris) that the pain was due to acute saturnine meningo-encephalitis. This condition was also especially noticed by Dr. Jefferis Turner in his series of cases of lead poisoning in children in Queensland,[1] and by Dr. Lockhart Gibson in his series also in children.[2] At the time of the convulsions all the reflexes were present and slightly exaggerated. After a few days the knee-jerks became weaker and remained so for several weeks, gradually recovering. There was marked right ankle clonus, which disappeared after six weeks.

*Ocular Symptoms.*—Pupils were always equal, though in the early part of the illness they were dilated and sluggish. After the convulsions, and when consciousness had returned, double convergent strabismus was noticed; this was due to complete paralysis of left external rectus and paresis of right. This paralysis of the external recti appears to have been first noticed by Lockhart Gibson, and published by him in a paper he wrote in 1897 in the *Australasian Medical Gazette.* In addition, there was at first sight general amblyopia, which very soon disappeared, leaving only a left temporal amblyopia, the outer half of the field of vision of left eye being quite cloudy. This eventually cleared up. There was no optic neuritis. Hare says that frequently in transient cases there are no ophthalmoscopic changes to be made out.

*Treatment.*—The only treatment when medicine could be taken was small doses of iodide of potassium, it was not deemed advisable to give large doses for fear of liberating too much free lead in the system, and so aggravating the symptoms in the acute stage; an ice bag at that period gave considerable relief.

[1] *Brit. M. J.*, 1909, i, 895. [2] *Ibid.*, 1908, ii, 1488.

But there was no indication either for bleeding or injection of large quantities of artificial serum, as recommended by the French physicians.

